# Diagnostic Value of Circulating Chromogranin A for Neuroendocrine Tumors: A Systematic Review and Meta-Analysis

**DOI:** 10.1371/journal.pone.0124884

**Published:** 2015-04-20

**Authors:** Xin Yang, Yuan Yang, Zhilu Li, Chen Cheng, Ting Yang, Cheng Wang, Lin Liu, Shengchun Liu

**Affiliations:** 1 Department of Endocrine and Breast Surgery, The First Affiliated Hospital of Chongqing Medical University, Chongqing, China; 2 Department of Cardiovascular Medicine, The First Affiliated Hospital of Chongqing Medical University, Chongqing, China; University Hospital Llandough, UNITED KINGDOM

## Abstract

**Background:**

In previous decades, chromogranin A (CgA) has been demonstrated to be the most promising biomarker for the diagnosis of neuroendocrine tumors (NETs), but its diagnostic value is still controversial. This meta-analysis aimed to estimate the potential diagnostic value of circulating CgA for NETs.

**Methods:**

We collected relevant studies from several electronic databases as well as from reference lists. Diagnostic indices of CgA were pooled with random effects models. Pooled sensitivity, specificity, positive likelihood ratio (PLR), negative likelihood ratio (NLR), diagnostic odds ratio (DOR) and summary receiver operating characteristic (SROC) curves for the diagnosis of NETs were used to estimate the overall diagnostic efficiency.

**Results:**

Through a search strategy, 13 studies met the inclusion criteria and were included. These studies contained 1260 patients with NETs and 967 healthy controls in the total sample. As a result, the overall sensitivity, specificity and diagnostic odds ratio (DOR) were 0.73 (95% CI: 0.71 to 0.76), 0.95 (95% CI: 0.93 to 0.96) and 56.29 (95% CI: 25.27 to 125.38), respectively, while the summary positive likelihood ratio (PLR) and negative likelihood ratio (NLR) were 14.56 (95% CI: 6.62 to 32.02) and 0.26 (95% CI: 0.18 to 0.38), respectively. In addition, the area under the curve (AUC) of the circulating CgA in the diagnosis of NETs was 0.8962.

**Conclusions:**

These data demonstrate that circulating CgA is an efficient biomarker for the diagnosis of NETs with high sensitivity and specificity, which indicates that it may be helpful for the clinical management of NETs. However, further studies are needed to clarify this issue.

## Introduction

Neuroendocrine tumors (NETs) are a group of rare and heterogeneous neoplasms that are derived from cells throughout the nervous and endocrine systems. These tumors are widely distributed throughout the human body including the stomach, intestine, pancreas, adrenals, and thyroid, among other areas [1–3]. The particular characteristic that distinguishes NETs from other solid malignancies is that NETs are composed of specialized cells that have the ability to produce, store, and secrete bioactive amines and peptide hormones [4].The incidence of NETs is very low, which is approximately 5.25 cases per 100,000 people or 1 case per 1,000 malignancies. However, an analysis of the US Surveillance Epidemiology and End Results (SEER) database showed that the incidence of NETs in the past several decades has substantially increased, which might be because of enhanced knowledge and improved diagnostic techniques [5, 6]. NETs are broadly classified into two categories termed functional NETs or non-functional NETs according to whether these tumors give rise to a clinical syndrome [7]. Although the awareness of this tumor type has improved, the ability of clinicians to diagnose NETs is hampered by enormous challenges in clinical practice. On the one hand, functional NETs may be discovered when they are in the distinctive early stage, but they are often misdiagnosed on account of nonspecific and unpredictable symptoms [3, 8–10]. On the other hand, most non-functional NETs with no symptoms or just local symptoms are not frequently identified until they have progressed to an advanced state, by which time metastasis has already occurred [3, 8–10]. Therefore, the consideration of practice guidelines for the diagnosis of this heterogeneous tumor type is a severe and pressing issue.

Chromogranin A (CgA) is an acidic glycoprotein that belongs to the granin family and is present in the secretory dense-core granules that function in the storage of peptide hormones and catecholamines in all endocrine and neuroendocrine cells [11, 12]. During the past several decades, a growing body of evidence has demonstrated that CgA is released in abnormal amounts by many neoplastic neuroendocrine cells, which may affect the various components of the tumor stroma and contribute to the regulation of tumor growth and progression [13–15]. However, elevated circulating CgA levels have been confirmed to be a helpful biochemical marker for the diagnosis of various types of NETs [16–19], but its practicability for the clinical management of NETs is still debatable.

To discuss whether CgA could act as a valuable diagnostic biomarker for NETs, we conducted this systematic review and meta-analysis through the pooling of pertinent studies that were acquired from a number of electronic databases without any limitations. Our data revealed that CgA may be a promising and feasible biomarker for the diagnosis of NETs.

## Materials and Methods

### Data sources and search strategy

Without any restrictions in terms of language, year of publication and publication status, all relevant primary research studies published on or before October 27, 2014 that focused on the diagnostic value of CgA for NETs were searched from the following electronic databases: PubMed, Embase, Chinese National Knowledge Infrastructure (CNKI), Chinese Biomedical Literature Database (CBM), and Wan Fang Data. We also manually searched the references of the eligible studies. Our search strategy included “neuroendocrine tumors or neuroendocrine cancers or neuroendocrine carcinomas or neuroendocrine neoplasms”, “Chromogranin A or Chromogranin a or CgA or CHGA” and “diagnosis or screening or detection”.

### Inclusion and exclusion criteria

Two investigators (XY, YY) screened all relevant articles on the basis of titles and abstracts and then skimmed the full text for any reasonable eligibility. A full discussion that led to a consensus was necessary to resolve any disagreements. Studies were included if they met the following inclusion criteria: (1) The patients with NETs were diagnosed by histopathology, and the included control individuals were healthy without any type of cancer, cardiac, renal and inflammatory diseases or any other major diseases; (2) Diagnostic tests and all blood samples were collected before treatment; (3) The circulating CgA was assessed independently, and the studies featured the relevant data in a fourfold table with a definitive cut-off value; (4) Both the patients with NETs and the control individuals numbered more than 15 cases. If the studies fulfilled any of the following criteria, they was excluded: (1) reviews, case reports, commentaries, editorials, meeting abstracts and letters; (2) duplicate publications; (3) ineligible samples, including patients, control subjects and blood samples; and (4) insufficient data. We included only the latest or the most complete reports by some authors who may have authored studies with overlapping populations or who may have had multiple publications. In addition, if the relevant articles were likely to contain the diagnostic information in the supplementary data or if the data were not shown, we contacted the author for the needed data.

### Data extraction

The 13 included studies were examined independently by two reviewers (XY, YY), and the following data were extracted: first author, publication year, country where the studies were conducted, number of patients and controls, patient characteristics, study design, assay method of the biomarkers, cutoff values and raw data in a fourfold table format. If disagreements arose, a consensus was reached after a full discussion with a third senior reviewer. We also asked the authors for any missing data.

### Quality assessment

A few publications have reported that the Quality Assessment of Diagnostic Accuracy Studies 2 (QUADAS-2) is widely recognized as a redesigned and improved tool. The QUADAS-2 consists of 4 key domains that are supported by signaling questions to aid judgment on the risk of bias, including “patient selection”, “index test”, “reference standard” and “flow and timing;” simultaneously, the QUADAS-2 rates the risk of bias and applicability concerns as “high”, “unclear” or “low” and involves studies in which the reference standard consists of follow-up [20]. Therefore, we used QUADAS-2 to assess the quality of the eligible studies.

### Statistical analysis

All statistical analyses in our meta-analysis were performed by Meta-DiSc and STATA 12.0 statistical software [21]. We reviewed the eligible studies and extracted the value of the diagnostic indices, including true positives, false positives, true negatives and false negatives. Before we embarked on this analysis, we used a Spearman correlation analysis to quantify the heterogeneity due to the threshold effect among the included studies. If no heterogeneity caused by the threshold effect was detected, raw data from each study were incorporated to acquire a pooled sensitivity, specificity, positive likelihood ratio (PLR), negative likelihood ratio (NLR) and diagnostic odds ratio (DOR), as well as their 95% confidence intervals [95% CI]. Meanwhile, we used statistical software to generate the summary receiver operator characteristic curve (SROC) and to simultaneously calculate the area under the curve (AUC). The sensitivity, specificity, PLR, NLR, and DOR of CgA for the diagnosis of NETs are presented as forest plots. Furthermore, the Cochran-Q method and the test for the inconsistency index (I^2^) were used to assess the non-threshold effect. As a result, a low p-value (≤0.05) and a high I^2^ value (≥50%) exposed the presence of heterogeneity caused by a non-threshold effect. When a non-threshold effect existed, we explored the sources by meta-regression analysis. For publication bias, all included studies were assessed by Deeks funnel plot with STATA 12.0 statistical software; the plots were expected to have a symmetrical funnel shape when publication bias was absent.

## Results

### Data selection

**Fig 1:**
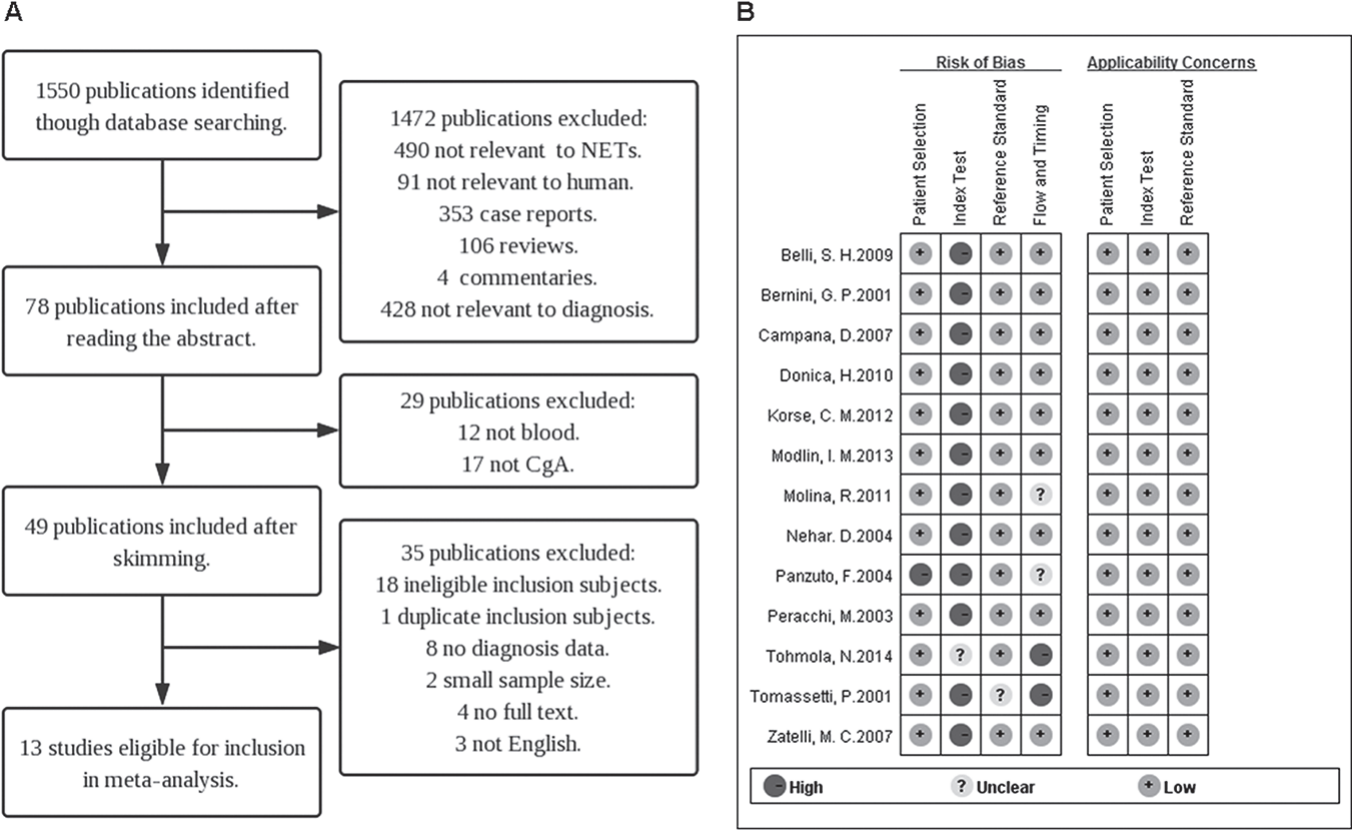
The flow diagram and the quality assessment. A) **The flow diagram.** This diagram shows the process of study selection. Finally, 13 eligible studies were included in our meta-analysis. B) **The quality assessment of the included studies by QUADAS-2.** This assessment summarizes “risk of bias” and “applicability concerns” through judging each domain in each included study and shows the major biases concentrated upon the “index text” and lesser biases that were related to the “flow and timing”.

As the flow diagram (Fig 1A) shows, through our literature search strategy, we originally obtained 1550 studies from electronic databases and reference lists. According to the titles and abstracts, 463 publications were excluded: 353 case reports, 106 reviews and 4 commentaries. In addition, 490 studies that focused on tumors other than NETs, 91 studies that involved non-humans subjects, and 428 studies that were irrelevant to the diagnosis were also excluded. The full-text versions of the remaining 78 articles were then obtained. Of these, 29 studies were excluded because the samples that were analyzed were not blood or because the biomarkers did not include CgA. Eventually, 49 studies that were perceived as potentially eligible were retrieved for an examination of the full text. After further analysis, an additional 16 studies were removed for the following reasons: data were absent (8 studies), the included subjects were duplicates (1 study), publication was not in English (3 studies), and the full text was not available (4 studies). According to our inclusion and exclusion criteria, 20 additional publications should have been excluded: 2 had small sample sizes and 18 had included ineligible patients and control individuals. Thus, 13 high-quality cohort studies with the informed consent of all participants met the inclusion criteria and were included for further analysis in this systematic review and meta-analysis [22–34].

### Study characteristics

All of these 13 included studies were published from 2001 to 2014 and had accumulated a total of 1260 patients with NETs and 967 healthy controls. Histopathology was recognized as the gold standard for the diagnosis of NETs. The study characteristics, such as first author, year of publication, country where the study was conducted, number of cases, mean age, assay method, cut-off value, true positive, false positive, false negative and true negative are listed in Table 1. Eight studies measured the serum or plasma levels of CgA by ELISA (enzyme-linked immunosorbent assay), and three of the thirteen used an RIA (radioimmunoassay). The remaining two studies used an IRMA (immunoradiometric assay). The sample size varied from 15 to 282 patients. With the exception of three studies that did not mention the age of the patients, the mean age of the patients ranged from 53 to 64 years of age. Furthermore, we list additional data about the NETs patients in Table 2, including the primary location and stage of NET, and the conditions that could result in an increase of CgA, such as whether the patients suffered from the cardiac, renal and inflammatory diseases or used the proton pump inhibitors. There were 4 studies that made no mention of the all above conditions, and the other 5 studies just lacked the information of proton pump inhibitors.

**Table 1:** Main characteristics of the studies included in this meta-analysis.

First author	Year	Country	Patients/ controls	Mean or median Age (yr)	Assay type	Cut-off values	True positive (TP)	False positive (FP)	False negative (FN)	True negative (TN)
Tohmola, N.	2014	Finland	41/26	64	RIA	6 nmol/L	21	4	20	22
Modlin, I. M.	2013	USA	81/94	NA	ELISA	19 U/L	26	1	55	93
Korse, C. M.	2012	The Netherlands	280/282	60	RIA	87 μg/l	193	14	87	268
Molina, R.	2011	Spain	66/52	NA	ELISA	60 ng/ml	55	0	11	52
Donica, H.	2010	Poland	41/15	58	ELISA	18 U/l	29	2	12	13
Belli, S. H.	2009	Argentina	119/39	53	RIA	2.8 nmol/L	110	0	9	39
Zatelli, M. C.	2007	Italy	81/129	58.5	ELISA	16 U/l	68	19	13	110
Campana, D.	2007	Italy	170/48	59	ELISA	18–19 U/l	145	2	25	46
Panzuto, F.	2004	Italy	68/24	53	ELISA	22 U/l	57	9	11	15
Nehar, D.	2004	France	124/50	53	IRMA	100 μg/l	82	0	42	50
Peracchi, M.	2003	Italy	61/50	54.6	ELISA	20 U/l	56	0	5	50
Bernini, G. P.	2001	Italy	48/130	NA	IRMA	100 ng/ml	36	0	12	130
Tomassetti, P.	2001	Italy	80/28	55	ELISA	17 U/l	45	0	35	28

**Table 2:** The details of NETs patients included in the meta-analysis.

First author (year)	Primaty location	Stage	Diseases that elevated CgA	Use of PPIs
Tohmola, N. (2014)	Gut	NA	NA	NA
Modlin, I. M. (2013)	GEP	NA	NA	NA
Korse, C. M. (2012)	GEP (137), lung/thorax (42), Others/unknown(101)	Local: 59; Metastases:221	NA	NA
Molina, R. (2011)	GEP*, lung*, others/ unknown*	Local: 21; Metastases: 45	Without this diseases	NA
Donica, H. (2010)	GEP(28), lung(6), liver(1), bladder(1), ovary(1), unknown(4)	Local:11; Metastases: 30	NA	NA
Belli, S. H. (2009)	GEP	Local: 35; Metastases: 84	Without this diseases	Not use
Zatelli, M. C. (2007)	GEP	NA	Without this diseases	Not use
Campana, D. (2007)	GEP*, lung*	Local: 75; Metastases: 95	Without this diseases	Not use
Panzuto, F. (2004)	GEP(57), others(11)	Local: 40; Metastases: 28	Without this diseases	Not use
Nehar, D. (2004)	GEP	Local: 27; Metastases: 97	Without this diseases	NA
Peracchi, M. (2003)	GEP	Local: 33; Metastases: 28	Without this diseases	NA
Bernini, G. P.(2001)	GEP(19), thyroid(3), lung(6), adrenal(20)	NA	Without this diseases	NA
Tomassetti, P. (2001)	GEP	NA	Without this diseases	NA

*: The studies did not mention the detail numbers. NA: Not available. The data of the NETs patients were not obtained from the studies.

### Quality assessment

We assessed the quality of the eligible studies using the QUADAS-2 quality assessment tool. As shown in Fig 1B, we found that the major biases of these eligible studies are concentrated within the “index text” because the interpretation of the index test always occurs after knowledge of the reference standard, and thus, the potential for this bias is related to the subjectivity of the authors. Furthermore, the cut-off value in a portion of the studies was not specified, which may optimize the test performance to some extent. However, all of the 13 included studies are of upper-middle quality.

### Heterogeneity and threshold effect

**Fig 2:**
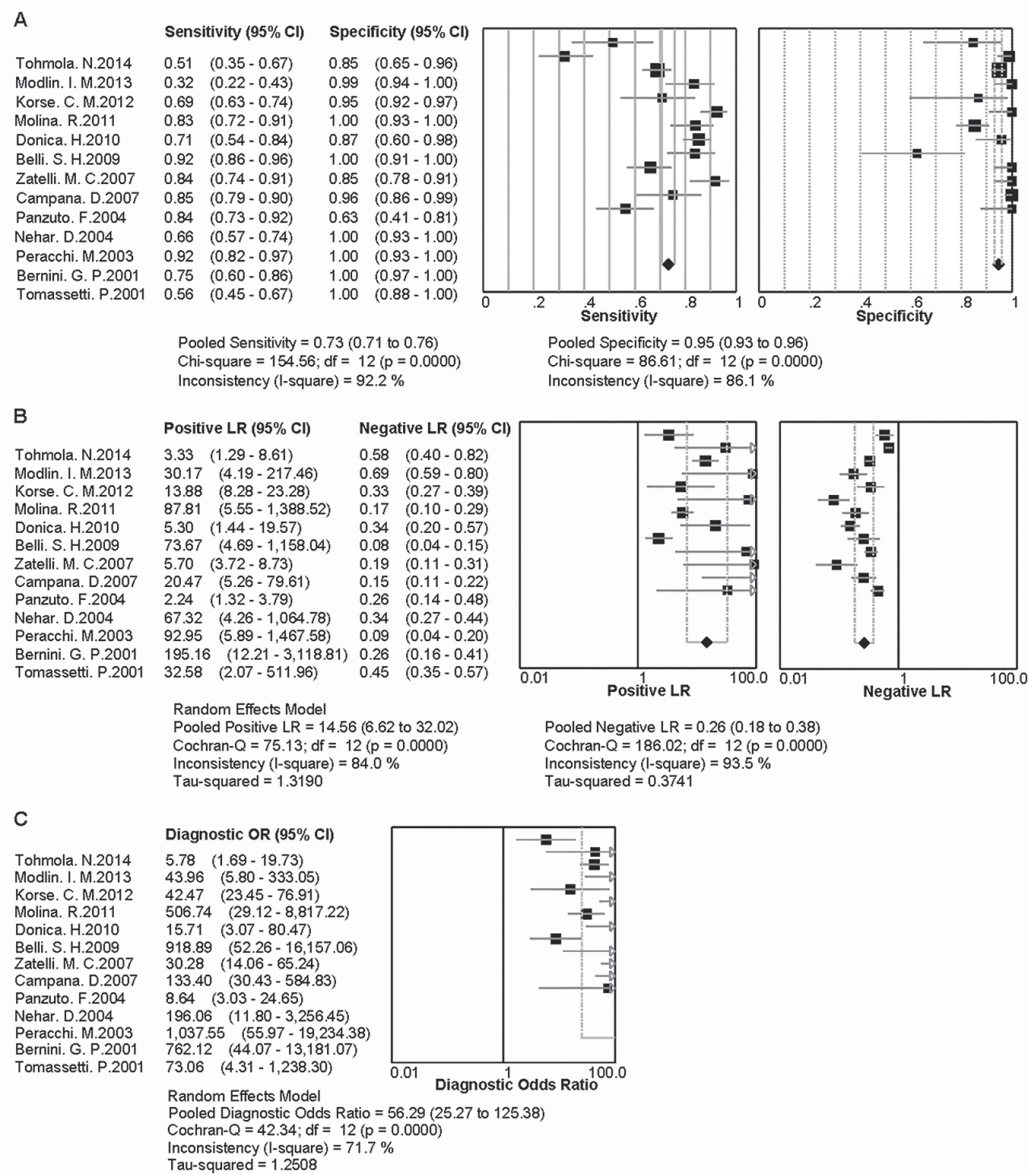
The forest plots show the pooled diagnostic indices of CgA for NETs. The heterogeneity caused by the non-threshold effect is quantified by inconsistency (I^2^). Because this heterogeneity exists, a random effects model was used to pool these data. The point efficiencies from each study are shown as squares, and the pooled efficiencies are shown as diamonds. The degree of freedom is abbreviated as df. As shown, A) the pooled sensitivity and specificity are 0.73 (95% CI: 0.71 to 0.76) and 0.95 (95% CI: 0.93 to 0.96), respectively. B) the pooled PLR and NLR are 14.56 (95% CI: 6.62 to 32.02) and 0.26 (95% CI: 0.18 to 0.38), respectively. C) the pooled DOR is 56.29 (95% CI: 25.27 to 125.38).

An examination of the potential sources of heterogeneity is indispensable for any meta-analysis before the pooling the data from the included studies into summary assessments. [35]. The determination of the heterogeneity within the studies is crucial to the comprehension of the potential factors that have a great effect on accuracy assessments and on the appraisal of the appropriateness of statistical pooling of the diagnostic accuracy from the various studies [21]. One of the primary and important causes of heterogeneity in the diagnostic accuracy of tests is the threshold effect. To assess whether the heterogeneity of CgA from the threshold effect exists in the included studies, we first used a Spearman test to calculate the correlation coefficient and *P* value between the logit of sensitivity and logit of 1-specificity. As a result, the Spearman correlation coefficient was -0.055, and the *P* value was 0.858 (>0.05), which could exclude the heterogeneity caused by the threshold effect. Another important factor that may contribute to the heterogeneity among the studies is the non-threshold effect. In this meta-analysis, the inconsistency index (I^2^) was employed to quantify the heterogeneity from the non-threshold effect. The I^2^ values in the forest plots of the diagnostic indices were more than 50% (as shown in Fig 2), which suggested that heterogeneity caused by the non-threshold effect existed among these studies.

### Data analysis

**Fig 3:**
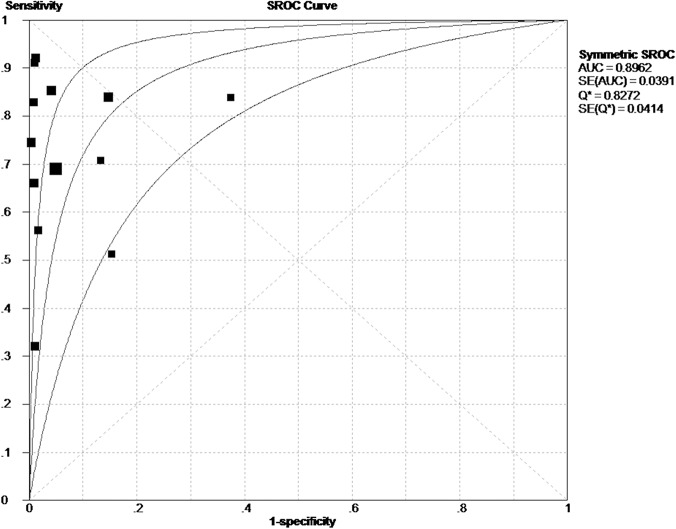
The summary receiver operating characteristic curves (SROC). Every square represents a study. The SROC curve is symmetric with the 0.8962 AUC, which intimates a higher diagnostic accuracy for the diagnosis of NETs.

In this meta-analysis, a random effects model was used to assess the overall test performance of CgA in the diagnosis of NETs due to the existence of heterogeneity from the non-threshold effect. The diagnostic indices of CgA including sensitivity, specificity, PLR, NLR and DOR of the 13 included studies are demonstrated by forest plots (Fig 2). As shown, the pooled sensitivity and specificity values of CgA were 0.73 (95% CI: 0.71 to 0.76) and 0.95 (95% CI: 0.93 to 0.96), respectively, in the diagnosis of patients with NETs (Fig 2A). The pooled PLR and NLR values for the diagnosis of NETs were 14.56 (95% CI: 6.62 to 32.02) and 0.26 (95% CI: 0.18 to 0.38), respectively (Fig 2B). Moreover, the summary DOR (Fig 2C) and the area under the SROC were 56.29 (95% CI: 25.27 to 125.38) and 0.8962 (Fig 3), respectively. All of these data suggested a higher diagnostic accuracy of CgA for the diagnosis of NETs. Among the included studies, three different assays were used to measure the level of circulating CgA, so we attempted to compare the diagnostic efficiency between the three different assays. As a result, the sensitivity was both 0.74 by the ELISA and RIA assays, while the sensitivity was 0.69 by the IRMA assays. The specificity was 0.93, 0.95 and 1.00 for the ELISA, RIA and IRMA assays respectively.

### Meta-regression

The reason that we performed a meta-regression was so that we could detect the existence of heterogeneity created by the non-threshold effect among the studies in the forest plot of the diagnostic indices (Fig 2). We attempted to uncover the source of the heterogeneity via an analysis of the study characteristics, such as country, year, mean age, assay type and sample size. Fortunately, the heterogeneity in our meta-analysis was closely related to the sample size (<30), and the *P* value is 0.0262.

### Publication bias

**Fig 4:**
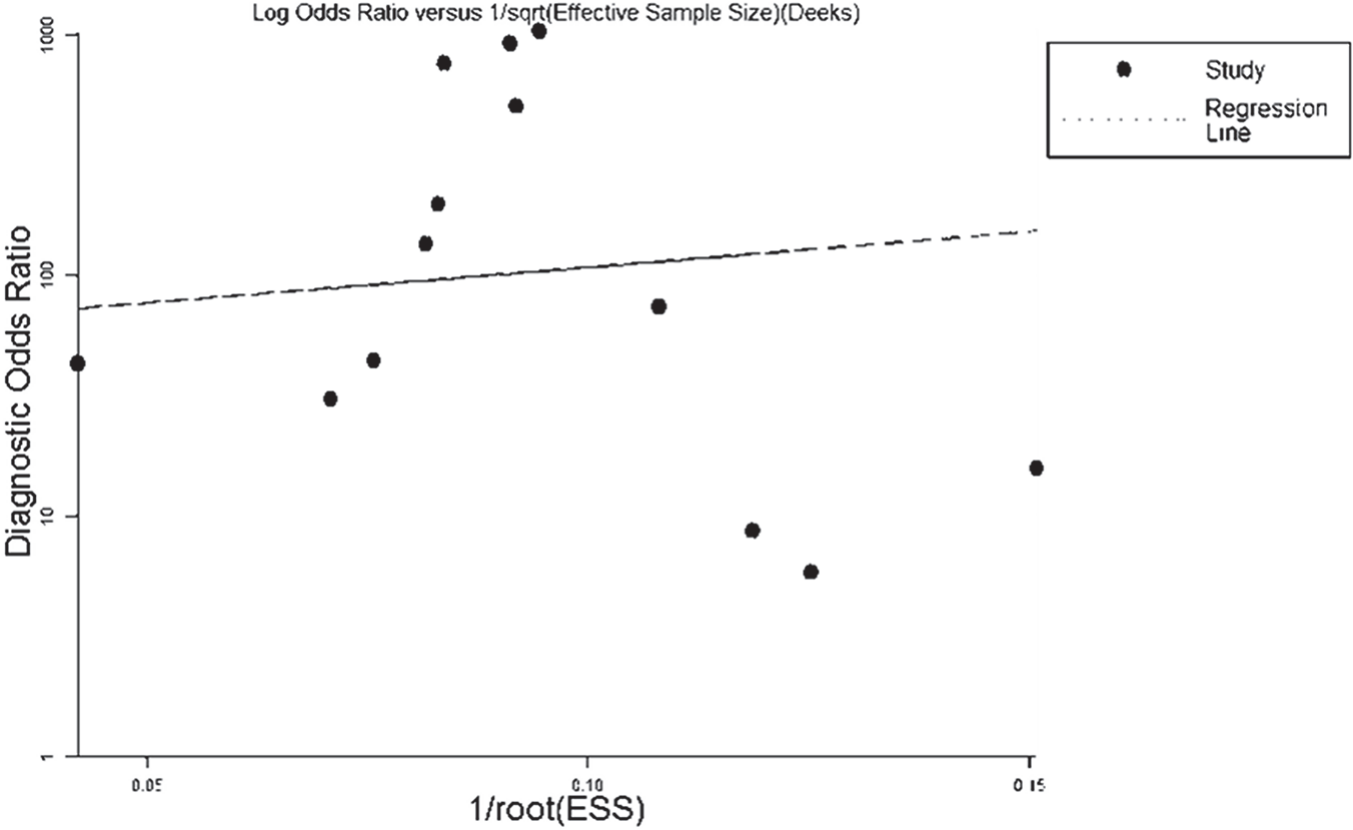
The funnel plots of publication bias. Every point represents one study, and the line is the regression line. The funnel shape is symmetrical, which indicates no publication bias.

It is recognized that publication bias is another important influential factor for overall diagnostic performance[36]. Therefore, a funnel plot was used to investigate whether all studies drew from a single population and to search for publication bias. The results showed a symmetrical funnel shape that indicated the absence of publication bias, which can be observed in Fig 4; the *P* value is 0.683.

## Discussion

Within several authoritative databases, we failed to find any studies that focused on CgA as a potential biomarker for the diagnosis of NETs by evidence-based evaluation during the past few decades. This foremost meta-analysis was performed to summarize the diagnostic performance of CgA for the diagnosis of NETs and to evaluate its prospect as a diagnostic biomarker for the detection of patients with NETs. Discrepant expression levels of CgA in the plasma or serum between the patients with NETs and the controls were apparent, and these differences were also statistically significant. Importantly, it has been acknowledged that the DOR is a diagnostic index that represents compactness between the diagnostic efficiency and the cases and has a remarkable test performance with an extremely high value [37]. In addition, AUC is another important index that is thought to represent overall diagnostic performance and have an optimal value when it is infinitely close to 1 [38]. As a conclusion of our meta-analysis, the CgA discriminated NETs from controls and yielded a DOR of 56.29 (95% CI: 25.27 to 125.38), an AUC of 0.8962 with a summary sensitivity of 0.73 (95% CI: 0.71 to 0.76), and a specificity of 0.95 (95% CI: 0.93 to 0.96). This supports the potential of a higher diagnosis value of CgA as a noninvasive detection tool.

We also compared the diagnostic efficiency between the three different assays, the results shown the sensitivity was 0.74, 0.74 and 0.69 for the ELISA, RIA and IRMA assays, and the specificity was 0.93, 0.95 and 1.00 for the ELISA, RIA and IRMA assays respectively. Although there was some difference between the three different assays, we thought this difference was lack of significance due to the difference of the studies numbers between the three assays. There were 8 studies that used the ELISA assays, but just 3 and 2 studies used the RIA and the IRMA assays respectively. However, any assays employed for CgA measurement displayed similar positive results that were in agreement with overall diagnostic efficiency.

For any meta-analysis or systematic review, the exploration of the potential influencing factors of heterogeneity is indispensable before the pooling of the raw data of the included studies into summary assessments to raise the accuracy [35]. Fortunately, heterogeneity that was generated by the threshold effect is absent in our meta-analysis. We attempted to expound the causes of heterogeneity by meta-regression by reason of the existence of heterogeneity from the non-threshold effect. Conclusively, among those that were related to the study level co-variables, such as country, year, mean age, assay type and sample size, the heterogeneity has a serious potential to be interrelated with uneven sample size. Another critical risk is the source and selection of studies. For any meta-analysis, publication bias is known as an important risk that might adversely affect the reliability of the conclusions. One of the common sources of publication bias is that the sampling is always restricted to published studies because published studies tend to present positive conclusions [39, 40]. However, publication bias is absent in our analysis, which might be because of our inclusion of a smaller number of primary studies.

Retrospectively, the name for these rare tumors, which are characterized by malignant histological type but less malignant behavior including a clear boundary and slow development, has changed since they were discovered in 1869. Initially, these tumors were named “carcinoids”, but subsequently this term was deemed inaccurate. Much later, *Feyrter* (1938) proposed the concept of a diffuse neuroendocrine system (DNES) that might be the origin of carcinoid tumors [41, 42]. In 1968, *Pearse* grouped the various neuroendocrine cells that belong to the unifying amine precursor uptake and decarboxylation (APUD) system, and soon after, the term “APUD cell tumors” appeared [43, 44]. Recently, neuroendocrine cells were redefined by *Langley*, *K*. [2], and the World Health Organization’s definition of neuroendocrine tumors have been used to describe this type of heterogeneous neoplasm [1]. With the progressive comprehension of NETs, the diagnostic techniques also continue to improve so that this disease can be better identified. In addition to some of the traditional methods, such as histopathology and medical imaging, the measurement of circulating tumor biomarkers has emerged as a responsible and noninvasive tool with which to monitor tumors [45]. Since 1984, when *O’Connor DT et al*. [46] first measured the circulating CgA in a patient with pheochromocytoma by the immunoassay method and demonstrated that CgA might be a potential biomarker of this tumor, many studies have evaluated its clinical impact in various neuroendocrine tumors [16–18, 47–49].

To highlight the superiority, we enumerate several obvious advantages of CgA as a potential useful biomarker for the diagnosis of NETs. First, the detection method of CgA is simple and inexpensive. CgA is physiologically released via exocytosis and may be stably detected in the blood; moreover, the blood sample is easy to obtain, and the immunology assay is easy to perform and control [50–52]. Meanwhile, the low cost of obtaining the blood sample and the noninvasive nature of detection make this process acceptable to patients. Second, CgA is expressed by both functioning and nonfunctioning tumors, and thus, the measurement of this biomarker is particularly meaningful [53]. In spite of the modified awareness and diagnostic techniques of NETs, plenty of misdiagnoses or delayed diagnosis still occur because of silent or feeble clinical manifestation, especially for nonfunctioning NETs [8]. Third, CgA is a more accurate and meaningful biomarker compared with 5-hydroxyindoleacetic acid (5-HIAA) and neuron-specific enolase (NSE). 5-HIAA, as the primary metabolite of serotonin, is sensitive to some serotonin-containing foods and drugs [54–56]. NSE is not a secretory protein and is located only in the cytoplasm; the presence of circulating NSE is thought to be related to a high death rate of cells with neuroendocrine differentiation, and in addition, both the sensitivity and specificity of NSE are lower than those of CgA [17, 57].

It is also undeniable that this meta-analysis has a few limitations in spite of the promising results. On the one hand, we failed to cover all potential studies. With regards to several studies with missing data or with no full text, we asked the authors for assistance. Unfortunately, some e-mails were lack of replies when we tried to contact the authors. On the other hand, small-study effects in our meta-analysis were unavoidable. We included several studies with sample sizes that were small due to a lack of NETs, and in recent decades, the clinical value of CgA has been investigated in NETs more thoroughly. With regards to this issue, further validations are necessary to strengthen our conclusion. Furthermore, some biases remained in this meta-analysis. Among the 13 studies, the cut-off value of CgA expression was reported to be selected from the ROC curve or from the upper limits of normal (ULN) for healthy individuals. This is generally considered to partly optimize the overall diagnostic efficiency. Additionally, none of the included studies unequivocally mentioned whether the investigators who interpreted the index test results were blinded to the reference test results; conversely, most of them knew the reference test results before the research. Thus, the test conclusion is prone to suffer from the subjectivity of the authors. Moreover, CgA have been demonstrated to be elevated in a variety of conditions, including neuroendocrine, cardiac, renal and inflammatory diseases, and some drugs like proton pump inhibitors. In our meta-analysis, the control subjects were healthy people without above conditions, but the NETs patients who suffered from the cardiac, renal and inflammatory diseases were not mentioned in 4 studies and who used proton pump inhibitors were not mentioned in 9 studies. These details were both absent in 4 studies. The lake of the details might have some impact on the overall diagnostic efficiency.

In conclusion, our data on the statistical method demonstrate that abnormally high circulating CgA levels are a characteristic feature of patients with NETs, a result that is in agreement with previous studies. At the same time, the detection of circulating CgA has a high sensitivity and specificity for the diagnosis of NETs. Consequently, circulating CgA can play an important role in the screening of NETs to diagnose and treat these tumors early. CgA could serve as a promising and noninvasive diagnostic biomarker of NETs in clinical practice after a validated large scale study.

## Supporting Information

S1 Checklist The PRISMA checklist. (DOC)
